# Factors Deciding Conservative or Intervention Treatment for Prostate Abscess: A Retrospective Case-Control Study

**DOI:** 10.3390/jpm13030484

**Published:** 2023-03-08

**Authors:** Yi-Huei Chang, Szu-Ying Pan, Chia-Yu Lin, Chi-Ping Huang, Chi-Jung Chung, Yung-Hsiang Chen, Wen-Chi Chen

**Affiliations:** 1 Department of Urology, China Medical University Hospital, Taichung 404327, Taiwan; 2 Department of Public Health, College of Public Health, China Medical University, Taichung 406333, Taiwan; 3 School of Medicine, College of Medicine, China Medical University, Taichung 404333, Taiwan; 4 Department of Medical Research, China Medical University Hospital, Taichung 404327,Taiwan; 5 Graduate Institute of Integrated Medicine, College of Chinese Medicine, China Medical University, Taichung 404333, Taiwan; 6 Department of Psychology, College of Medical and Health Science, Asia University, Taichung 413305, Taiwan

**Keywords:** prostate abscess, conservative treatment, intervention therapy, transurethral prostate drainage

## Abstract

Prostate abscess (PA) can lead to severe urosepsis and septic shock if not treated promptly. However, early diagnosis can be hindered by the declining incidence of PA, especially in developing countries and high-risk patients. Despite the prevalence of PA, there is currently a lack of well-established contemporary guidelines or treatment algorithms. This study aimed to review the etiology, pathophysiology, diagnosis, and treatment options for PA, as well as analyze the characteristics, background profiles of patients, and clinical course. Ultimately, the goal was to develop a personalized treatment strategy for patients with PA. This retrospective study examined 44 patients diagnosed with PA at a tertiary medical center between 2010 and 2020. The patients were divided into two groups based on their treatment: conservative treatment or intervention (transurethral resection of the prostate [TURP] or transurethral prostate drainage [TPD]). The study evaluated various factors, including patients’ background profiles, comorbidities, laboratory data, and PA size and volume. Complications of the interventions were also analyzed. No significant differences were found in basic data between the conservative treatment group (19 patients) and intervention group (25 patients; 20 for TURP, 5 for TPD). However, it was observed that single abscesses, size <2.2 cm, and prostate volume <48 cm^3^, may be suitable for conservative treatment. Patients with diabetes mellitus and human immunodeficiency virus should be monitored for thrombotic events. In addition, there was a significant difference in white blood count between the two groups (12.1 ± 7.0 vs. 17.6 ± 9.7 × 10^9^/L, *p* < 0.05). A subgroup analysis of the intervention group showed no significant difference in the risk of complications between TPD and TURP. Patients with poorly controlled diabetes mellitus and immunodeficiency are at a high risk of PA but are not indicated for surgical treatment. The PA profile, including number, size, volume, and percentage to prostate volume, should be considered when deciding on surgical intervention for patients with PA. Patients with higher leukocytosis may require surgical treatment. Overall, these findings can help guide the development of a personalized treatment strategy for patients with PA.

## 1. Introduction

Prostate abscess (PA) is a type of acute infection that affects the lower urinary tract [1,2]. Fortunately, the incidence of PA has decreased in recent years due to the widespread use of antibiotics. Nevertheless, patients with PA can still experience a range of distressing symptoms, including dysuria, frequency, urgency, and urinary retention [3]. Additionally, individuals with PA often present with fever and perineal pain. While broad-spectrum antibiotics are commonly used to combat infections, conservative treatment may fail to effectively resolve the abscess in some instances. In light of the emergence of antibiotic-resistant bacteria, atypical infections, and immunocompromised patients, managing PA has become increasingly challenging. In fact, a recent study by Ackerman and colleagues suggested that surgical drainage should be initiated promptly in such cases to prevent complications such as sepsis and septic shock [4]. Therefore, it is critical to further investigate the timing and indications for surgical treatment in patients with PA. Doing so may help healthcare professionals to optimize patient outcomes [5,6,7] and reduce the risk of severe complications associated with this condition.

The current state of PA treatment guidelines is lacking, and further research is needed in this area [8]. In a retrospective review of 18 patients with PA, Oshinomi et al. divided the treatment into two groups: conservative and drainage [9]. The use of imaging modalities is crucial for the accurate diagnosis and effective management of PA. Ultrasound, computed tomography (CT), and magnetic resonance imaging (MRI) are just a few of the imaging techniques available for this purpose. These imaging modalities can provide valuable information on the size, location, and number of abscesses in the prostate gland. In a study by Ackerman et al., it was found that abscesses larger than 30 mm are an indication for transurethral drainage, regardless of whether they are focal or multiple. However, the optimal approach to treating patients with urinary retention remains unclear, as this aspect was not investigated in the aforementioned research. Furthermore, CT imaging is particularly useful for accurately diagnosing PA, especially when differentiating it from other urinary tract infections. Given the rise in antibiotic-resistant strains of bacteria, prompt diagnosis and appropriate treatment initiation based on imaging findings are crucial for managing PA and reducing the risk of severe complications such as sepsis and shock [10]. Additional research in this area may provide valuable insights into optimizing PA treatment protocols and improving patient outcomes.

This retrospective study aimed to comprehensively evaluate the most effective treatment modalities for patients diagnosed with PA. In order to achieve this goal, the investigation conducted an extensive analysis of the patients’ medical history, comorbidities, therapeutic interventions, and clinical outcomes. The findings of this study carry substantial implications for clinical practice, furnishing crucial insights that can guide medical professionals in developing personalized treatment plans for patients afflicted with PA [11,12,13,14]. The results of this research have the potential to significantly enhance patient outcomes and, as a result, elevate the quality of care provided to individuals grappling with this condition.

## 2. Materials and Methods

### 2.1. Patients and Study Design

Prior to commencement, the study underwent a rigorous review process and received ethical approval from the Institutional Review Board of China Medical University Hospital (IRB: CMUH 112-REC1-014), Taichung, Taiwan. This board is responsible for ensuring that all research involving human subjects adheres to the highest ethical standards and that the rights and welfare of study participants are protected. The approval of the IRB indicates that the study was designed and conducted in a manner that prioritized the safety and wellbeing of the participants while producing scientifically sound results. It is important to note that obtaining ethical approval from an IRB is a critical step in any research involving human subjects, as it indicates that the study meets the ethical guidelines and standards set forth by regulatory bodies. This commitment to ethical conduct not only protects the rights of participants, but also helps to establish trust and confidence in the research findings among the broader scientific community and the general public [15,16,17].

From January 2010 to Mach 2020, a total of 44 male patients diagnosed with a PA were recruited from China Medical University Hospital. The recruitment process involved a thorough evaluation of patients presenting with suspected PA for clinical symptoms and laboratory data. The presenting symptoms of patients included typical lower urinary tract symptoms such as dysuria, urgency, frequency, and urinary retention, as well as fever or perineal pain. Laboratory data revealed the presence of an infection, including leukocytosis and pyuria. The diagnosis of PA was made based on a combination of clinical presentation, laboratory findings, and imaging studies, such as suprapubic ultrasound and CT scans.

Patients with small and single abscesses who were in a stable clinical condition and did not have sepsis were treated conservatively with an intravenous administration of broad-spectrum antibiotics, according to the results of cultures. On the other hand, patients with larger and multiple abscesses or those with sepsis required surgical intervention, typically after a 2-week course of intravenous antibiotics had failed. It is worth noting that the decision to opt for conservative or surgical treatment was made after careful consideration of the patient’s clinical condition and imaging findings.

Of the 44 patients recruited, 19 were successfully treated with conservative management, indicating the effectiveness of this approach for certain cases of PA. The results of this study contribute to the growing body of knowledge on the optimal management of PA, especially in the context of emerging antibiotic-resistant strains of bacteria and the need for individualized treatment approaches.

The interventional treatment group for patients with PA consisted of 5 patients who received transurethral prostate drainage (TPD) and 20 patients who underwent transurethral resection of the prostate (TURP) [18,19]. The size of the abscess and the size of the prostate were important factors in determining the appropriate treatment approach. TPD was used for small abscesses (less than 1 cm^3^), while TURP was reserved for larger abscesses and prostate size. Comorbidities such as DM, hypertension, CAD, HIV infection, and cirrhosis of the liver were analyzed to determine their impact on treatment outcomes.

Laboratory data, such as complete blood count, urine and blood culture; C-reactive protein; and HbA1c, were also analyzed to assess the severity of the infection and the overall health of the patients.

**Figure 1:**
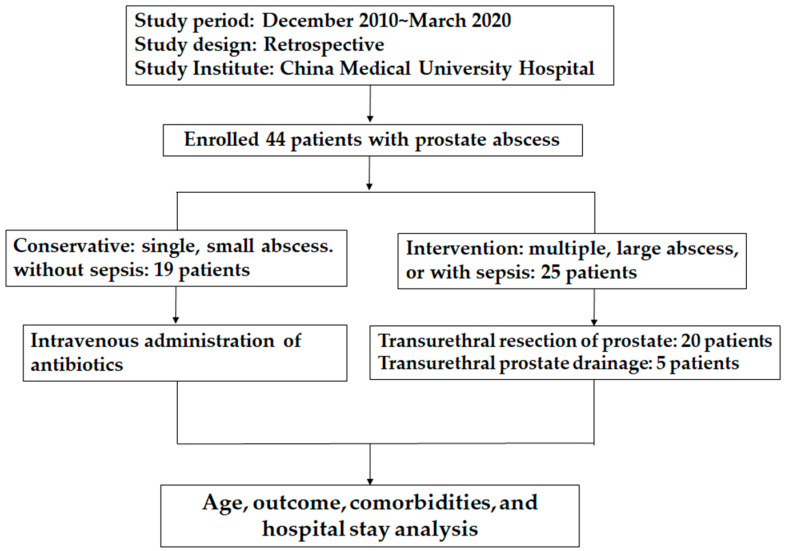
Flow chart of retrospective analysis of the patients with a prostate abscess.

The flow chart depicted in Figure 1 provides a visual representation of the study design and patient inclusion criteria. The retrospective analysis of patients with PA involved a thorough review of their medical records, including clinical presentation, laboratory data, imaging studies, and treatment outcomes. The study was approved by the IRB of China Medical University Hospital, ensuring that all ethical considerations were taken into account. Overall, this study provides valuable insights into the optimal management of PA and the factors that influence treatment outcomes.

### 2.2. Statistical Assay

SPSS version 25 was used for all statistical analyses (IBM Inc., Armonk, NY, USA). The Student’s *t* test was used for comparisons of independent numeric parameters between the two study groups. The Mann–Whitney U test was used for non-parametric analysis between the two groups. A *p* value < 0.05 was considered statistically significant.

## 3. Results

**Figure 2:**
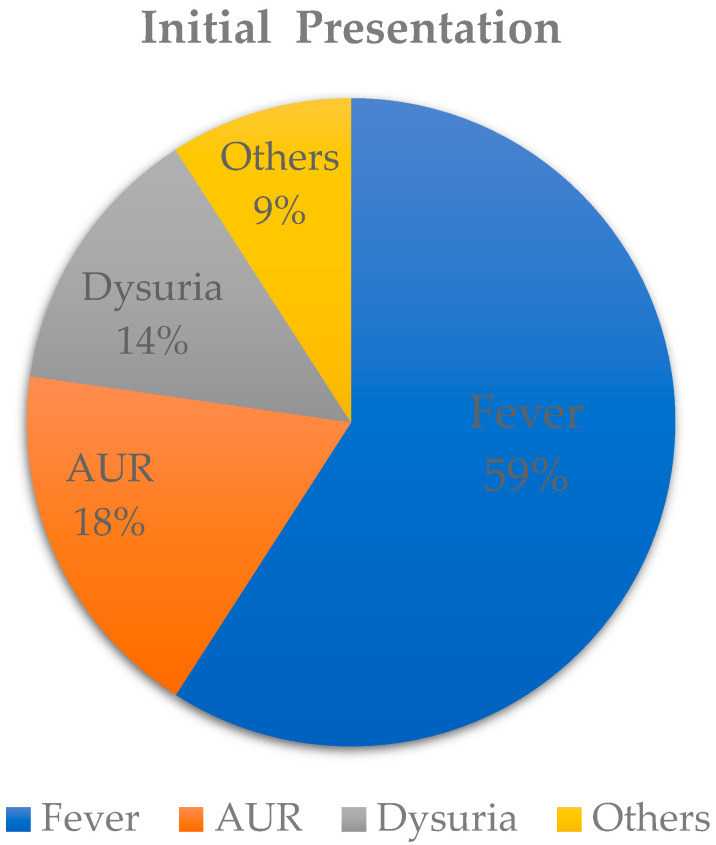
Initial symptoms and comorbidities of patients with a prostate abscess.

Of the 44 patients, 26 (59.1%) initially presented with fever (Figure 2). The second most common presentation was acute urinary retention (nine patients, 18.2%), and six patients (13.6%) had symptoms of dysuria. There was no significant difference in average age between the two groups (Table 1).

All patients in the study achieved successful treatment outcomes with symptomatic relief and were subsequently discharged. Notably, the intervention group exhibited a considerably prolonged average hospital stay in comparison to the conservative group (18.5 vs. 11.6 days, *p* = 0.02). The prevalence of diabetes mellitus was higher in the conservative group, with 15 patients (78%) being affected, as opposed to 11 patients (44%) in the intervention group (*p* = 0.02). Conversely, no significant differences were observed in the incidence of other comorbidities, including cirrhosis, hypertension, coronary artery disease, and human immunodeficiency virus, between the two study groups, as presented in Table 2.

The white blood cell (WBC) count in the intervention group was significantly higher than that in the conservative group (*p* = 0.04). There were no significant differences in laboratory data between the two groups, including urine WBC count, C-reactive protein, HbA1c, and serum creatinine (Table 3). Regarding the PAs, the size, number, and volume of the PAs were significantly larger in the intervention group (Table 1). The prostate volume in the conservative group was 48 ± 14 cm^3^, compared to 71 ± 27 cm^3^ in the intervention group (*p* = 0.003).

## 4. Discussion

This study aimed to establish a personalized treatment algorithm for patients with PA by analyzing the records of 44 patients admitted to China Medical University Hospital from 2010 to 2020. The analysis included patients’ background profiles, clinical courses, laboratory studies, PA size and volume, outcomes, and intervention complications. The patients were categorized into conservative (*n* = 19) and intervention (*n* = 25) groups based on the treatment they received. Among the patients, 59.1% had diabetes mellitus (DM), while other comorbidities included hypertension, coronary artery disease (CAD), liver cirrhosis, and thrombotic events. A small proportion of patients (6.8%) had HIV infection.

Our analysis suggests that a personalized treatment approach should be adopted for patients diagnosed with PA. Specifically, patients presenting with smaller PA sizes (less than 2.2 cm in diameter) or low prostate volume (<48 cm^3^) may be suitable candidates for conservative management. Conversely, patients presenting with larger abscesses or elevated white blood cell counts may benefit from intervention to prevent the progression of the disease and the consequent accumulation of pus in the prostate. As such, we recommend that healthcare professionals carefully evaluate each patient’s unique clinical characteristics before deciding on the most appropriate treatment strategy. In this study, both the conservative and intervention groups had similar basic characteristics, with median ages of 57.1 and 59.6 years, respectively, consistent with the typical age range for PA occurrence. The average hospital stay for the intervention group was significantly longer than that of the conservative group (18.5 vs. 11.6 days), which is in line with previous research by Alnadhari et al. [20]. The longer stay in the intervention group may be attributed to more advanced disease and severe symptoms requiring a longer recovery time, as all patients received medical treatment followed by intervention treatment with TURP or TPD.

Even though the incidence of PA has decreased with the use of broad-spectrum antibiotics, PA remains a challenge and has a high mortality rate in high-risk groups, including those who are immunocompromised and those with DM. The initial presentation of PA is usually unspecific, and includes fever; acute urinary retention; dysuria; urgency; frequency; the sensation of incomplete voiding and tenesmus, which can therefore be misdiagnosed as urethritis; urinary tract infection; and acute or chronic bacterial prostatitis. If the diagnosis is delayed or neglected, PA can progress to a lethal condition with a mortality rate ranging from 1% to 16% [21].

The treatment of PA can generally be divided into conservative and interventional. Conservative treatment entails the use of broad-spectrum parenteral antibiotics, which are usually given during the hospital course, followed by specific antibiotics based on culture and sensitivity results. The most commonly used intervention treatments are TURP and TPD, both of which have the advantages of a fast recovery, lower recurrence rate, and reduced hospitalization time.

The early diagnosis and prompt treatment of PA is crucial [22,23], since most PAs develop from acute or chronic bacterial prostatitis. The initial presentation of a PA is usually non-specific, including systemic symptoms such as fever (59%), acute urinary retention (18%), and dysuria (14%); thus, it can be misdiagnosed as urethritis, urinary tract infection, acute or chronic bacterial prostatitis, benign prostatic hyperplasia, or urinary obstruction. Therefore, further physical examinations, laboratory studies, or imaging studies may be needed for the differential diagnosis [5,24]. According to the literature, over 95% of patients with PA have a painful digital rectal examination [4], with the presence of a palpable fluctuant prostate in 16% to 88% of patients [25,26]. Laboratory results of PA usually mimic a urinary tract infection, with the presence of leukocytosis, pyuria, and bacteria. Since such positive physical examination findings and laboratory results cannot distinguish a PA from prostatitis, Ha et al. suggested that patients with acute prostatitis who do not respond to treatment after 48 h should be evaluated for a possible PA [21].

There can be a correlation between the organisms obtained from abscesses, urine cultures, and blood cultures, but it ultimately depends on the specific case. In general, if there is an infection in the urinary tract, it is possible for bacteria to travel up the urinary tract and cause a kidney or prostate abscess [27]. In such cases, the organisms obtained from the abscess may be similar or identical to those found in the urine culture. If blood cultures were obtained, they can provide valuable information about the presence of bacteremia (bacteria in the bloodstream) and the potential spread of infection to other parts of the body. Positive blood cultures can indicate the presence of the same bacteria that caused the abscess or urinary tract infection, but it is not always the case. Sometimes, different bacteria can cause infections in different parts of the body. Ultimately, the correlation between the organisms obtained from the abscess, urine cultures, and blood cultures will depend on the specific case and the individual’s unique medical history and circumstances. It is important to work closely with a healthcare provider to determine the most appropriate diagnostic and treatment plan for any suspected infection.

Concerning prostatic abscesses (PAs), our study observed significant differences in terms of number, size, volume, percentage, and volume between two patient groups. Patients with a larger PA volume (mean ± SD: 25.4 ± 21.7 cm^3^) necessitated intervention treatment. Our findings suggest that patients diagnosed with a PA exceeding 2 cm in diameter should consider intervention treatment, while those with a smaller PA diameter may be eligible for conservative treatment, although clinical symptoms must be taken into account. Medical interventions may include antibiotic therapy, transrectal ultrasound-guided aspiration or drainage, and surgical excision. Conservative management may consist of antimicrobial agents, analgesics, and close monitoring of the patient’s condition. Further research is needed to explore optimal treatment strategies for PAs of various sizes and clinical presentations.

A normal prostate gland is approximately 3 × 3 × 5 cm in size or has a volume of 25 mL [28]. The larger the prostate volume, the higher the likelihood it could develop into BPH and cause symptoms similar to lower urinary tract symptoms, such as increased frequency, urgency, nocturia, dysuria, starting urination, weak urine stream, or dribbling at the end of urination. The symptoms of BPH can be difficult to distinguish from other urinary obstruction diseases, and BPH itself is a risk factor for PA.

In the current study, the median prostate volume in the conservative group was 48 ± 18 cm^3^, compared to 71 ± 27 cm^3^ in the intervention group. The WBC count results also showed different profiles between the two groups, with 12,100/μL in the conservative group and 17,600/μL in the intervention group, and the difference was statistically significant. These findings are consistent with the clinical features of PA and show that purulent formation within the prostate may exceed the penetration of antibiotics, and that a surgical approach is essential in such cases.

Fever of unknown origin is a common presentation in men, and imaging studies such as computed tomography (CT), magnetic resonance imaging (MRI), and transrectal ultrasound are often used to identify potential sources. In some medical centers, transrectal ultrasound is utilized to evaluate the presence of prostatic abscesses. Additionally, in certain institutions, ultrasound-guided transperineal or transrectal needle aspiration is conducted with favorable outcomes, which may be followed by an intracavitary injection of antibiotics. However, our study did not involve transperineal or transrectal aspiration or injection of the abscess. Future research may investigate the effectiveness and safety of such interventions in managing prostatic abscesses, including potential complications such as bleeding, infection, or urinary dysfunction. Further, the development of standardized protocols for the diagnosis and management of prostatic abscesses would aid in providing optimal patient care and outcomes.

In the realm of diagnostic imaging, micro-ultrasound (MUS) is an innovative technique that displays potential in enhancing the quality of ultrasonic imaging. MUS is a real-time imaging modality characterized by high spatial resolution, which has recently been introduced in the field of urology. MUS offers a multitude of benefits over traditional ultrasound imaging, including the ability to discern subtle changes in prostatic tissue, augment the visualization of the prostate capsule and surrounding tissue, and heighten the detection of small lesions. Additionally, MUS has the potential to guide biopsy procedures with greater accuracy, reducing the need for repetitive biopsies and mitigating patient discomfort. Future studies may explore the utility of MUS in the diagnosis and management of prostatic abscesses, potentially improving outcomes for patients.

This novel tool aims to describe the current evidence regarding the application of MUS for the diagnosis and detection of benign and even malignant lesions. Research has demonstrated high sensitivity and specificity for the diagnosis of prostate cancer with MUS. Given its ability to provide high-resolution images, MUS may also prove useful for the diagnosis of prostatic abscess in the future. The high spatial resolution of MUS allows for precise identification of the location and size of abscesses, which can facilitate the selection of an appropriate management strategy. Furthermore, MUS-guided drainage procedures can potentially reduce the risk of complications and improve patient outcomes. To establish the diagnostic and therapeutic utility of MUS in the management of prostatic abscesses, further studies are necessary. Additionally, studies comparing the accuracy of MUS with other imaging modalities in detecting and characterizing prostatic abscesses would be valuable in determining the most effective diagnostic and management strategies for patients [29].

PA is an uncommon cause of urinary tract infections (UTIs), especially in individuals who have compromised immune systems or abnormalities in their urinary tract. While conservative treatment approaches may be effective in managing mild cases, surgical intervention may be necessary in more severe cases. Determining the optimal timing for surgical intervention is a major clinical challenge. Various drainage procedures have been suggested as potential interventions to reduce the duration of antibiotic therapy, shorten hospital stays, and improve voiding function. The effectiveness of these procedures, however, may be influenced by factors such as the patient’s age, comorbidities, and severity of infection. Therefore, a comprehensive evaluation of each patient’s condition is necessary to determine the appropriate treatment strategy for managing UTIs caused by PA [30].

This study has some limitations, including its retrospective design, nonrandomized participant assignment, and small sample size. These limitations suggest that more rigorous research with larger sample sizes is needed to confirm these findings and broaden our understanding of the topic. Furthermore, this study was conducted in a single center, which may limit the generalizability of the results to other populations. In addition, the use of different antibiotics and surgical techniques among patients may have influenced the outcomes, leading to potential bias. The lack of long-term follow-up data also limits our ability to draw conclusions about the long-term outcomes of different treatment modalities. Future studies with longer follow-up periods are needed to investigate the long-term efficacy and safety of different treatment approaches for PA. Despite these limitations, this study provides valuable insights into the management of PA and may serve as a starting point for future research in this field.

## 5. Conclusions

PA is a serious condition that requires careful consideration of various factors for treatment. Patients with poorly controlled diabetes or immunodeficiency are at a higher risk of developing PA. Treatment for PA should be based on the severity of the abscess, associated comorbidities, and response to antibiotics. Interventional treatments, such as TPD or TURP, may be necessary for larger abscesses or those that do not respond to antibiotics. Early recognition and appropriate management are crucial for preventing complications and improving outcomes. Additionally, imaging plays a vital role in the diagnosis and management of PA, and various imaging modalities such as ultrasound, CT, and MRI can assist in determining the size, location, and number of abscesses in the prostate gland. The development of antibiotic-resistant strains of bacteria highlights the importance of prompt and accurate diagnosis and the initiation of appropriate treatment. While the findings of this study suggest that conservative treatment with antibiotics can be effective for small and single abscesses in stable patients, further research is needed to determine the optimal treatment approach for patients with urinary retention. Despite the limitations of this study, the results provide valuable insights into the management of PA and can inform medical professionals in developing personalized treatment plans for patients with this condition. Ultimately, improving the quality of care provided to individuals suffering from PA will require continued research efforts and advances in diagnostic and treatment modalities.

## Figures and Tables

**Table 1:** Patients’ profile and abscess size.

	Conservative	Intervention	*p* Value
Patient number	19	25	
Age (years)	57.1	59.6	0.61
Hospital days	11.6	18.5	0.02
Abscess			
Number	1.3 ± 0.5	1.8 ± 0.7	0.025
Size (cm)	2.2 ± 0.6	3.1 ± 1.0	0.001
Volume (cm^3^)	7.5 ± 6.9	25.4 ± 21.7	0.001
Percentage (%)	14.2 ± 9.1	32.3 ± 18.3	<0.001
Prostate volume (cm^3^)	48 ± 14	71 ± 27	0.003

Mann–Whitney U test, Student’s *t* test.

**Table 2:** Comorbidities of the two study groups.

Comorbidities	Conservative (*n* = 19)	Intervention (*n* = 25)	*p* Value
Diabetes	15 (78%)	11 (44%)	0.02 *
Hypertension	5 (26.3%)	4 (16%)	0.40
CAD	5 (26.3%)	3 (12%)	0.22
Cirrhosis	2 (10.5%)	6 (24%)	0.25
HIV	1 (5.3%)	2 (8%)	0.77

CAD: coronary artery disease, HIV: human immunodeficiency virus. * *p* < 0.05, Mann–Whitney U test, Student’s *t* test.

**Table 3:** Laboratory data of the two study groups.

Laboratory	Conservative (*n* = 19)	Intervention (*n* = 25)	*p* Value
WBC (average)	12.1 ± 7.0	17.6 ± 9.7	0.04 *
Urine WBC	598.0 ± 439.8	627.0 ± 434.3	0.86
CRP	9.3 ± 11.8	10.3 ± 10.7	0.41
Serum creatinine	1.16 ± 0.51	1.47 ± 1.26	0.46

WBC: white blood cell count, CRP: C-reactive protein, HbA1c: glycated hemoglobin. * *p* < 0.05, Mann–Whitney U test, Student’s *t* test.

## Data Availability

All data are available upon request to the corresponding author.
